# Screening and validating of endogenous reference genes in *Chlorella* sp. TLD 6B under abiotic stress

**DOI:** 10.1038/s41598-023-28311-x

**Published:** 2023-01-27

**Authors:** Yongshun Zhou, Fanze Meng, Kai Han, Kaiyue Zhang, Jianfeng Gao, Fulong Chen

**Affiliations:** College of Life Sciences, Shihezi University, Shihezi, Xinjiang 832000 People’s Republic of China

**Keywords:** Plant molecular biology, Plant stress responses, Abiotic

## Abstract

*Chlorella* sp. TLD 6B, a microalgae growing in the Taklamakan Desert, Xinjiang of China, is a good model material for studying the physiological and environmental adaptation mechanisms of plants in their arid habitats, as its adaptation to the harsh desert environment has led to its strong resistance. However, when using real-time quantitative polymerase chain reaction (RT-qPCR) to analyze the gene expression of this algae under abiotic stress, it is essential to find the suitable endogenous reference genes so to obtain reliable results. This study assessed the expression stability of 9 endogenous reference genes of *Chlorella* sp. TLD 6B under four abiotic stresses (drought, salt, cold and heat). These genes were selected based on the analysis results calculated by the three algorithmic procedures of geNorm, NormFinder, and BestKeeper, which were ranked by refinder. Our research showed that *18S* and *GTP* under drought stress, *18S* and *IDH* under salt stress, *CYP* and *18S* under cold stress, *GTP* and *IDH* under heat stress were the most stable endogenous reference genes. Moreover, *UBC* and *18S* were the most suitable endogenous reference gene combinations for all samples. In contrast, *GAPDH* and *α-TUB* were the two least stable endogenous reference genes in all experimental samples. Additionally, the selected genes have been verified to be durable and reliable by detecting *POD* and *PXG3* genes using above endogenous reference genes. The identification of reliable endogenous reference genes guarantees more accurate RT-qPCR quantification for *Chlorella* sp. TLD 6B, facilitating functional genomics studies of deserts Chlorella as well as the mining of resistance genes.

## Introduction

Algae are widely distributed in freshwater, seawater, humid soil, desert and extreme environments. The order microalgae is widely used and studied in bioenergy, medical treatment, wastewater treatment and food^1–3^. Deserts and arid lands cover one-third of the Earth’s land surface and are biologically challenging ecosystems. Microalgae are a vital component of desert biocrusts in different regions of the world^4–7^, and the adaptation of desert microalgae to harsh desert environments has led to a diversity of adaptive mechanisms (including ecological, physiological, morphological structural, genetic, etc.). Desert algae have higher photosynthetic efficiency than freshwater algae and stronger resistance to drought, salt, ultraviolet (UV), and temperature extremes^8–12^. Therefore, desert microalgae are better materials for studying biological and environmental adaptation mechanisms and mining resistance genes. *Chlorella* sp. TLD 6B is a single celled green alga isolated from biological crusts in Taklimakan Desert^13^. It has the advantages of high photosynthetic efficiency and stress resistance^14^. Thus, it is important to elucidate the molecular mechanism of the rapid growth and stress adaptation of chlorella for its utilization and protection of soil desertification. Based on the above reasons, our group generated a full-length transcriptome database of *Chlorella* sp. TLD 6B using a combination of single-molecule real-time (SMRT) sequencing (unpublished) and Illumina sequencing^15^.

Currently, gene expression studies have become an important tool for revealing gene functions and plant stress resistance molecular mechanisms. Real-time quantitative polymerase chain reaction (RT-qPCR) has turned into a standard method for studying gene expression due to its high sensitivity, reproducibility, specificity, and high throughput^16–19^. To effectively control the differences between the initial templates of each sample and ensure the reliability of RT-qPCR results, using endogenous reference genes for standardized analysis^20–23^ is a must. It has been found that some reference genes are stably expressed in different tissues and at different developmental stages, however, their expression stability can be altered to varying degrees upon treatment with some abiotic stresses^24–26^. Currently, there are still no endogenous reference genes that can be applied to more than one species, and even for the same species, hardly any reference genes are universal to examine multiple abiotic stress treatments. To this end, it is necessary to screen for stable endogenous reference genes in specific species. To date, many researches have been reported to differentiate this kind of genes in various species, such as Schima superba^27^, Isodon rubescens^28^, Miscanthus sacchariflorus^29^, Suaeda glauca^30^, Taxus spp^31^, Glehnia littoralis^32^. Although some papers^33–38^ have also studied on those reference genes in algae, given to the differences among microalgae species, these genes cannot be directly used as the reference genes of desert Chlorella to study its gene expression. Therefore, stable endogenous reference genes in desert Chlorella have to be screened for further studies.

**Figure 1:**
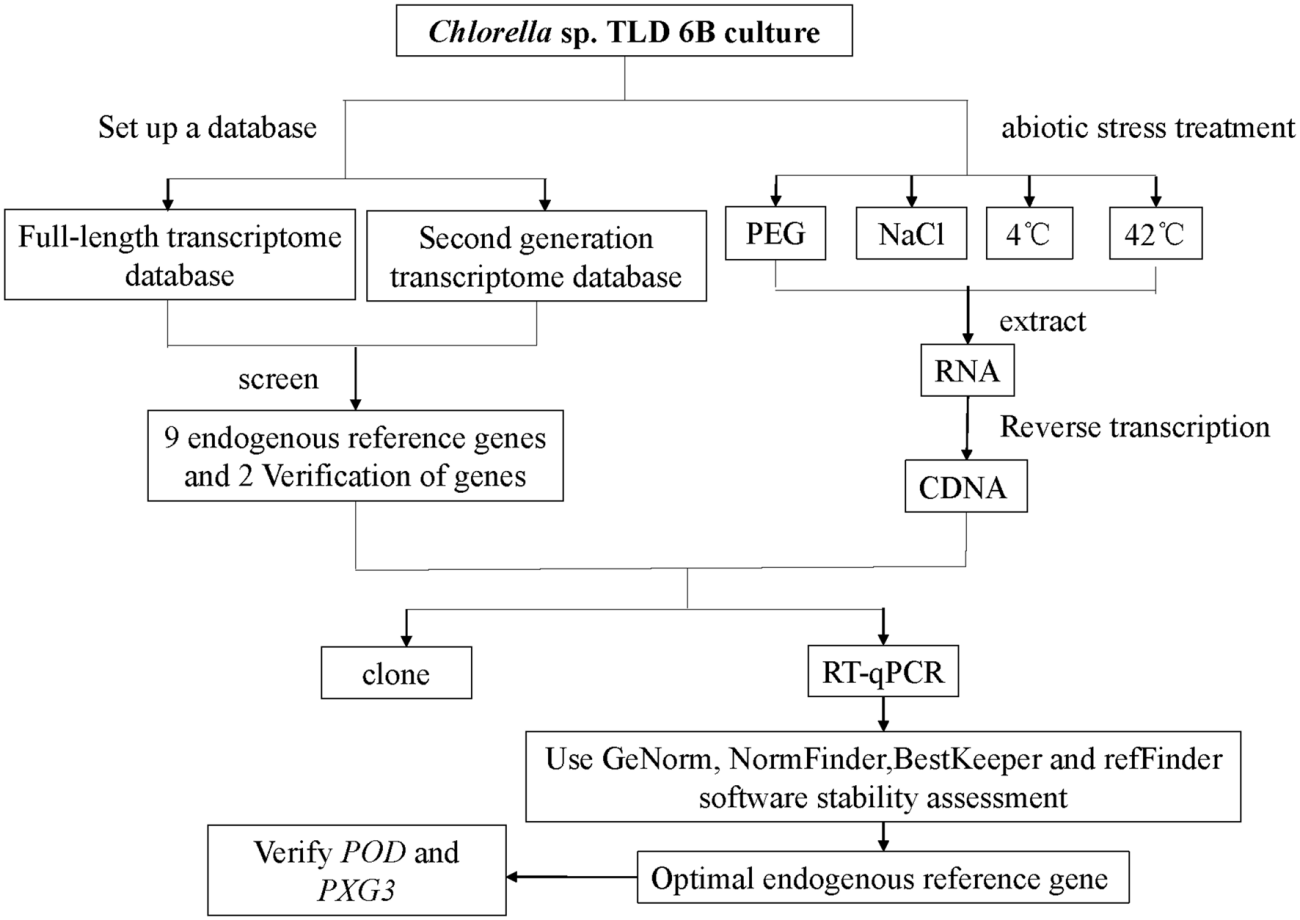
Experimental framework.

The genomic information of desert microalgae is unknown, which hinders the further study of its gene function. In this paper, based on transcriptome data^15^ of *Chlorella* sp. TLD 6B and reported reference genes in other species, we selected 18S rRNA (*18S*), Cyclophilin (*CYP*), Elongation factor 1α (*EF-1α*), Glyceraldehyde-3-phosphate Dehydrogenase (*GAPDH*), GTP binding protein (*GTP*), NADP-isocitrate dehydrogenase (*IDH*), Ubiquitin-conjugating enzyme (*UBC*), tubulin alpha chain (*α-TUB*), and tubulin beta chain (*β-TUB*) as endogenous reference genes^39–42^. Peroxidase (*POD*)^43^ and Peroxygenase 3 (*PXG3*)^44^ stress inducible genes were used to verify the stability of the above genes under different stress conditions. For *Chlorella* sp. TLD 6B after drought, salt, cold, heat treatment, we used GeNorm^45^, NormFinder^46^, bestkeeper^47^ and RefFind^48^ software to analyze the stability of endogenous reference genes (Fig. 1). Reliable endogenous reference genes were selected to ensure more accurate RT-qPCR quantification of *Chlorella* TLD 6B. This study not only promoted the functional genomics of Chlorella desert, but also laid a foundation for the exploration of resistance genes and the molecular mechanism of *Chlorella* sp.TLD 6B adaptation to extreme desert environment.

## Results

### Primer design

After the comparison and validation of *POD*, *PXG3* and the 9 endogenous reference genes in NCBI, primers were designed with Primer Premier 5 according to the principle of RT-qPCR primer design (Table 1). The PCR products of nine candidate reference genes were detected by agarose gel electrophoresis, and each gene obtained a single specific desired size band (Supplementary Fig. S1).

**Table 1:** Genes and primer pairs of the endogenous reference gene used for RT- qPCR.

Gene symbol	Description	Accession ID	Primer sequence	Amplification size
*18S*	18S ribosomal RNA	Dech-6B_transcript_48114	F:CTGGATCAGCGTTCGTCG	170
			R:TGATTTCAGCGGAGGTCAG	
*CYP*	Cyclophilin	Dech-6B_transcript_14505	F:TGGATGAGACGCACCTAGTTGTG	110
			R:CTGGAAGAAGGGGCTGTTGG	
*EF1α*	Elongation factor 1α	Dech-6B_transcript_9145	F:GATGGCTGAGGTGGTGTTTGAGC	145
			R:TTACGCCTTGGGGGTGGTCT	
*GAPDH*	Glyceraldehyde-3-phosphate Dehydrogenase	Dech-6B_transcript_44570	F:CCCTCTCTTGCCCTTTCTCCGT	156
			R:GGTCTTGGGTTGTGCTTGCTGC	
*GTP*	GTP binding protein	Dech-6B_transcript_17752	F:TTTCCGCCCGCTTGGAAT	118
			R:GCGTGATGATGGAGGTTTTGC	
*IDH*	NADP-isocitrate dehydrogenase	Dech-6B_transcript_39928	F:CTGGAAGAAGCCGATTGTGGTG	154
			R:GCCCTTGAAGTCGTAGATGGTGAA	
*UBC*	Ubiquitin-conjugating enzyme	Dech-6B_transcript_34066	F:CGGGAGATGCCGACTACTGC	107
			R:CAGCCCTGTCCTGGTTTTCTAA	
*α-TUB*	Tubulin alpha chain	Dech-6B_transcript_45761	F:CGGCTGCTTGAGGCGTCGTT	111
			R:GCTTGCCCAATGTGGATAGAAATAA	
*β-TUB*	Tubulin beta chain	Dech-6B_transcript_29059	F:GCAACAACTGGGCCAAGGG	198
			R:TCATGCGGTCGGGGTACTCC	
*PXG3*	Peroxygenase 3	Dech-6B_transcript_13983	F:AGCGACAGCGAGATCTT	146
			R:CAACCCAGCCGACAAAG	
*POD*	Peroxidase	Dech-6B_transcript_57541	F:CGCAGTTTGTTGCACTGC	107
			R:CCACCATCTCCTGGTCGT	

### Expression levels and variation of the endogenous reference genes

**Figure 2:**
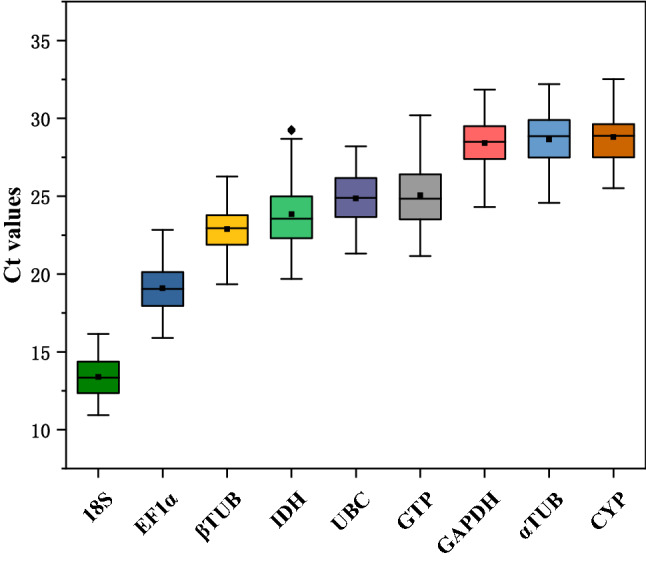
Ct value of endogenous reference genes across all treatments under four abiotic stresses. The line on each box represents the median. The little box represents the mean. The lower and upper edges of boxes show the 25th and 75th percentiles, respectively, and the circles represent outliers.

RT-qPCR was performed to detect the expression levels of nine genes. The results showed that the Ct values of all endogenous reference genes under different treatments ranged from 10.9 ~ 32.5 (Fig. 2), among which the average Ct value of *18S* was 13.4, with the highest expression and the lowest variation range (10.9 ~ 16.2) among the endogenous reference genes. Still, the highest coefficient of variation was 9.7%. *α-TUB*, *CYP*, and the mean Ct values of *α-TUB*, *CYP*, and *GAPDH* were 28.6, 28.8 and 28.4, respectively, with the lowest expression and coefficients of variation of 6.3, 5.8 and 5.7%, respectively, with *GAPDH* having the smallest coefficient of variation among all samples. The mean Ct values of *EF1α*, *UBC*, *β-TUB*, and *IDH* were 19.1, 24.9, 22.9, and 23.8 respectively.

### Stability assessment of endogenous reference genes using GeNorm software

**Figure 3:**
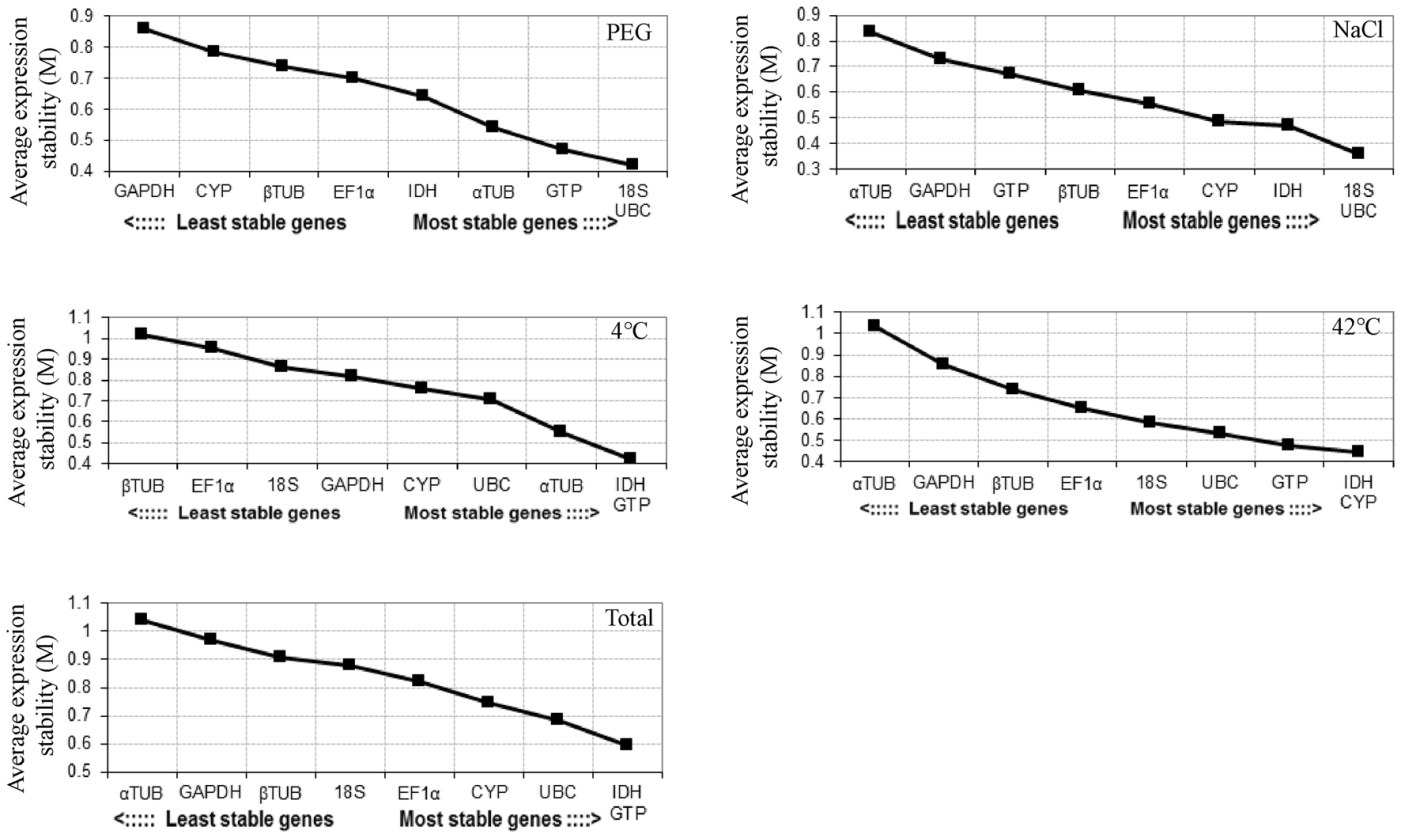
The expression stability values (M) of nine endogenous reference genes were determined by GeNorm software. The most stable and least stable genes are on the right and left. *PEG* PEG-6000 drought treatment, *NaCl* 200 mM NaCl salt treatment, *42 °C* 42 °C high temperature treatment, *4 °C* 4 °C cold treatment, *Total* Pooled samples from all treatments.

GeNorm program was used to calculate the stability value (M value) of gene expression of nine endogenous reference genes, and these genes were ranked. M < 1.5 was used as the threshold for excluding stable genes, and the lower the M value, the higher the stability. According to this criterion, *UBC* and 18S were the most stable endogenous reference genes in the PEG and NaCl treatment, *GTP* and *IDH* were the most stable endogenous reference genes in the 4 °C treatment and all samples, *CYP* and *IDH* were the most stable endogenous reference genes in the 42 °C treatment (Fig. 3). *α-TUB* and *GAPDH* were the least stable endogenous reference genes in NaCl, 42 °C and Total, *β-TUB* and *EF1α* were the least stable endogenous reference genes in the 4 °C treatment, and *GAPDH* and *CYP* were the least stable endogenous reference genes in PEG. The geNorm programme calculates the gene expression stability value (M value) and ranks the reference Genes.

**Figure 4:**
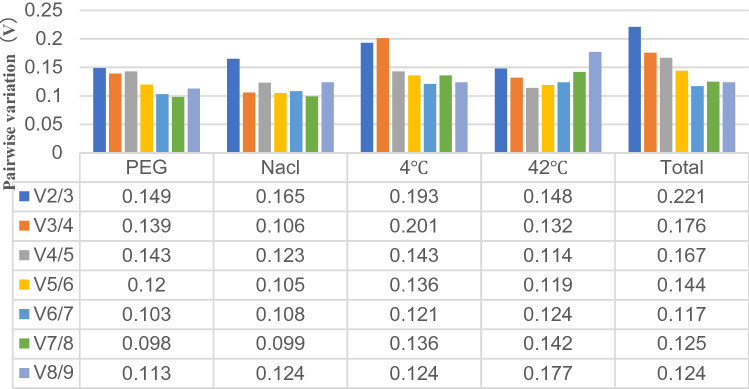
Pairwise variation (V) of endogenous reference genes, as calculated by GeNorm software. V_n_/V_n+1_ values were used to determine the optimal number of endogenous reference genes. A cut-off of 0.15 (Vn value) is usually applied. *PEG* PEG-6000 drought treatment, *NaCl* 200 mM NaCl salt treatment, *42 °C* 42 °C high temperature treatment, *4 °C* 4 °C cold treatment, *Total* pooled samples from all treatments.

Pairwise variation (V_n_/V_n+1_) values were calculated using the GeNorm software program to determine the optimal number of endogenous reference genes required for qRT-PCR to normalize target gene expression levels. Small variations between V_n_/V_n+1_ and V_n+1_/V_n+2_ indicated that the addition of another endogenous reference gene had no significant effect on normalization. The V_n_/V_n+1_ value of 0.15 was considered to be the threshold for deciding whether to add an endogenous reference gene was considered as the threshold value. The results showed (Fig. 4) V_2/3_ values below 0.15 for PEG (0.149) and 42 °C (0.148), indicating that only two suitable endogenous reference genes are required in PEG and 42 °C stress. V_3/4_ values below 0.15 for NaCl (0.106) and three endogenous reference genes are needed in NaCl stress. However, a threshold of 0.15 should not be considered a strict criterion, and some reports found higher threshold values for V_n_/V_n+1_^43–45^. The data showed a slight variation between V_2/3_ (0.193) and V_3/4_ (0.201) in the 4 °C samples, suggesting that three endogenous genes are required in 4 °C stress. There was a slight variation in the total samples between V_3/4_ (0.176) and V_4/5_ (0.167), indicating the need for 4 endogenous reference genes in all samples.

### Stability assessment of endogenous reference genes using NormFinder software

The stability values of the nine endogenous reference genes were calculated using the NormFinder software program, with lower values representing higher stability. *GTP* was most stable in PEG (0.222) and 42 °C (0.165); *IDH* was most stable in NaCl (0.140); *CYP* was most stable at 4 °C (0.217); and *UBC* was most stable in all samples (Total. 0.347) were the most stable (Table 2). The stability levels of the endogenous reference genes generated using NormFinder were slightly different from those generated using GeNorm. The endogenous reference genes that were the most stable in NormFinder analysis in PEG, NaCl, 4 °C, 42 °C and Total (*GTP*, *IDH*, *CYP*, *GTP* and *UBC*) had their stability as third (*GTP*, *IDH*), fifth (*CYP*) and third (*GTP*, *UBC*) in GeNorm analysis, respectively.

**Table 2:** Determination of stability values of endogenous reference genes using NormFinder software.

Rank	PEG	NaCl	4 °C	42 °C	Total
1	*GTP *(0.222)	*IDH *(0.140)	*CYP *(0.217)	*GTP *(0.165)/	*UBC *(0.347)
2	*18S* (0.295)	*18S* (0.175)	*18S* (0.388)	*18S* (0.239)/	*CYP *(0.390)
3	*IDH *(0.336)	*UBC *(0.240)	*UBC *(0.390)	*IDH *(0.276)	*18S* (0.464)
4	*UBC *(0.352)	*CYP *(0.262)	*GTP *(0.488)	*UBC *(0.277)	*EF1α *(0.489)
5	*EF1α *(0.435)	*EF1α *(0.427)	*IDH *(0.515)	*CYP *(0.333)	*GTP *(0.514)
6	*β-TUB *(0.439)	*GTP *(0.452)	*α-TUB *(0.520)	*EF1α *(0.524)	*IDH *(0.601)
7	*α-TUB *(0.459)	*GAPDH *(0.522)	*GAPDH *(0.578)	*GAPDH *(0.661)	*β-TUB *(0.610)
8	*CYP *(0.540)	*β-TUB *(0.528)	*EF1α *(0.646)	*β-TUB *(0.760)	*GAPDH *(0.677)
9	*GAPDH *(0.690)	*α-TUB *(0.760)	*β-TUB *(0.754)	*α-TUB *(1.094)	*α-TUB *(0.761)

The higher the ranking, the stronger the stability.

*PEG* PEG-6000 drought treatment, *NaCl* 200 mM NaCl salt treatment, *42 °C* 42 °C high temperature treatment, *4 °C* 4 °C cold treatment, *Total* pooled samples from all treatments.

### Stability evaluation of the endogenous reference genes using BestKeeper software

The Ct values were calculated using BestKeeper software to evaluate the expression stability of the nine endogenous reference genes. The principle of determination was that the greater the correlation coefficient, the smaller the standard deviation and the coefficient of variation, the better the stability of the endogenous reference genes, and vice versa, the worse the stability. In PEG *β-TUB* (4.66 ± 1.08) was the most stable endogenous reference gene; in NaCl and in *CYP* (2.97 ± 0.84) was the most stable endogenous reference gene; in 4 °C *β-TUB* (3.84 ± 0.91) was the most stable endogenous reference gene; in 42 °C and Total *18S* (6.82 ± 0.86, 7.79 ± 1.04) was the most stable endogenous reference gene (Table 3). *18S* was ranked first in all treatments and was a relatively stable endogenous reference gene, while *α-TUB* was ranked second in all treatments and was a relatively unstable endogenous reference gene. For various stresses, the stability ranking of the endogenous reference genes generated by BestKeeper was different from that of GeNorm and NormFinder.

**Table 3:** Stability analysis of endogenous reference genes using BestKeeper software.

Rank	PEG	NaCl	4 °C	42 °C	Total
1 (CV ± sd)	*β-TUB* (4.66 ± 1.08)	*CYP* (2.97 ± 0.84)	*β-TUB* (3.84 ± 0.91)	*18S* (6.82 ± 0.86)	*18S* (7.79 ± 1.04)
2 (CV ± sd)	*CYP* (4.26 ± 1.19)	*18S* (6.91 ± 0.88)	*18S* (9.34 ± 1.26)	*β-TUB* (4.16 ± 0.90)	*β-TUB* (5.13 ± 1.17)
3 (CV ± sd)	*18S* (10.25 ± 1.37)	*GTP* (3.69 ± 0.88)	*EF1α* (6.46 ± 1.28)	*IDH* (4.11 ± 0.91)	*EF1α* ( 6.37 ± 1.21)
4 (CV ± sd)	*EF1α* ( 7.31 ± 1.39)	*UBC* (3.69 ± 0.88)	*CYP* (5.00 ± 1.50)	*EF1α* (5.35 ± 0.97)	*CYP* (4.33 ± 1.25)
5 (CV ± sd)	*IDH* (5.98 ± 1.39)	*IDH* (3.95 ± 0.91)	*UBC* (6.06 ± 1.56)	*CYP* ( 3.76 ± 1.05)	*GAPDH* (4.49 ± 1.27)
6 (CV ± sd)	*UBC* (5.87 ± 1.44)	*β-TUB* (4.03 ± 0.92)	*GAPDH* (6.02 ± 1.74)	*GTP* (4.47 ± 1.06)	*UBC* (5.33 ± 1.33)
7 (CV ± sd)	*GAPDH* (5.46 ± 1.51)	*EF1α* (4.95 ± 0.93)	*α- TUB* (6.99 ± 2.01)	*UBC* (4.49 ± 1.06)	*α- TUB* (4.87 ± 1.40)
8 (CV ± sd)	*GTP* (6.40 ± 1.57)	*GAPDH* (3.63 ± 1.00)	*GTP* (7.61 ± 2.02)	*GAPDH* (4.87 ± 1.35)	*GTP* (6.40 ± 1.60)
9 (CV ± sd)	*α-TUB* (6.40 ± 1.75)	*α-TUB* (5.87 ± 1.63)	*IDH* (8.26 ± 2.11)	*α-TUB* (5.61 ± 1.58)	*IDH* (6.87 ± 1.64)

The higher the ranking, the stronger the stability.

*CV and SD* coefficient of variation and standard deviation, respectively.

*PEG* PEG-6000 drought treatment, *NaCl* 200 mM NaCl salt treatment, *42℃* 42℃ high temperature treatment, *4℃* 4℃ cold treatment, *Total* pooled samples from all treatments.

### Ranking of endogenous reference genes using RefFinder software

The RefFinder software program is used to determine the overall ranking of endogenous reference genes. The program integrates GeNorm, NormFinder, BestKeeper and △Ct methods. The ranking order under PEG treatment is: *18S* > *GTP* > *UBC* > *IDH* > *β-TUB* > *EF1α* > *CYP* > *α-TUB* > *GAPDH*. Under NaCl treatment is: *18S* > *IDH* > *UBC* > *CYP* > *GTP* > *EF1α* > *β-TUB* > *GAPDH* > *α-TUB*. Under 4 °C treatment is *CYP* > *18S* > *UBC* > *GTP* > *IDH* > *β-TUB* > *α-TUB* > *EF1α* > *GAPDH*. At 42 °C, the sorting order was: *GTP* > *IDH* > *18S* > *CYP* > *UBC* > *EF1α* > *β-TUB* > *GAPDH* > *α-TUB*. At all treatments, the sorting order was: *UBC* > *18S* > *CYP* > *GTP* > *EF1α* > *IDH* > *β-TUB* > *GADPH* > *α-TUB* (Table 4). *18S* was most stable in PEG and NaCl; *CYP* was most stable in 4 °C; *GTP* was most stable in 42 °C; *UBC* was most stable in all samples. The *α-TUB* and *GAPDH* were unstable in all stress treatments.

**Table 4:** Identification of the most stable and least stable endogenous reference genes using RefFinder analysis.

Rank	PEG	NaCl	4 °C	42 °C	Total
1	*18S *(1.86)	*18S* (1.68)	*CYP *(2.11)	*GTP *(2.06)	*UBC *(2.06)
2	*GTP *(2.21)	*IDH *(1.97)	*18S* (3.03)	*IDH* (2.06)	*18S* (2.71)
3	*UBC *(3.13)	*UBC *(2.45)	*UBC *(3.31)	*18S *(2.51)	*CYP *(2.83)
4	*IDH *(3.87)	*CYP *(2.83)	*GTP *(3.36)	*CYP *(3.34)	*GTP *(3.76)
5	*β-T UB *(3.98)	*GTP *(5.24)	*IDH* (3.87)	*UBC *(4.28)	*EF1α *(3.94)
6	*EF1α *(4.95)	*EF1α *(5.44)	*β-TUB *(5.2)	*EF1α *(5.42)	*IDH *(4.24)
7	*CYP *(5.66)	*β-T UB *(6.70)	*α-TUB *(5.24)	*β-TUB *(5.47)	*β-TUB *(5.12)
8	*α-TUB *(6.84)	*GAPDH *(7.74)	*EF1α *(6.26)	*GAPDH *(7.48)	*GAPDH *(7.11)
9	*GAPDH *(8.45)	*α-TUB *(9.00)	*GAPDH *(6.48)	*α-TUB *(9.00)	*α-TUB *(8.45)

The higher the ranking, the stronger the stability.

*PEG* PEG-6000 drought treatment, *NaCl* 200 mM NaCl salt treatment, *42 °C* 42 °C high temperature treatment, *4 °C* 4 °C cold treatment, *Total* pooled samples from all treatments.

### Target gene expression and endogenous reference gene validation

**Figure 5:**
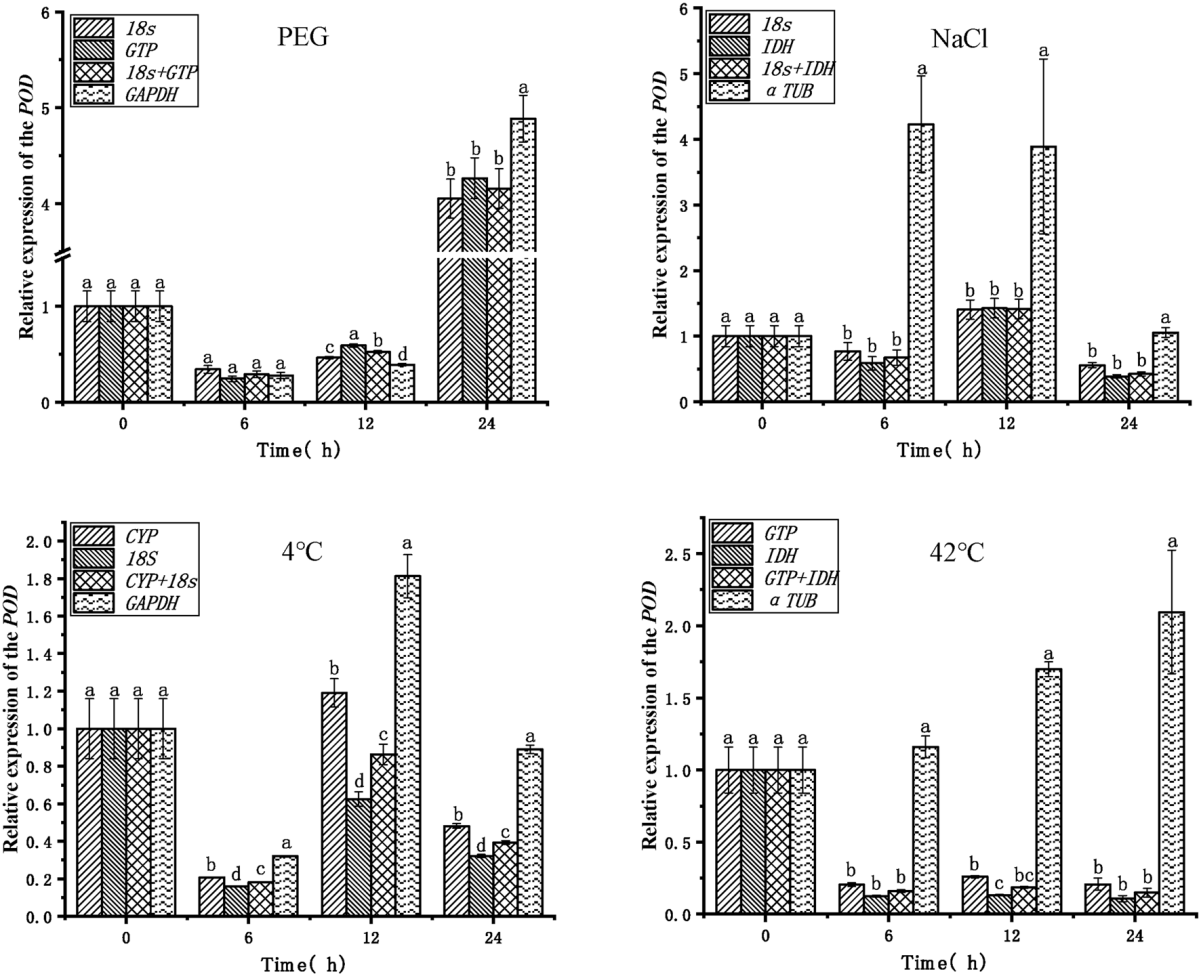
Relative expression of the *POD* target gene for validation of selected endogenous reference genes. *PEG* PEG-6000 drought treatment, *NaCl* 200 mM NaCl salt treatment, *4 °C* 4 °C cold treatment, *42 °C* 42 °C high temperature treatment. Different lowercase letters represent statistically significant differences as determined by one-way ANOVA (*P* < 0.05, Duncan’s multiple range tests).

**Figure 6:**
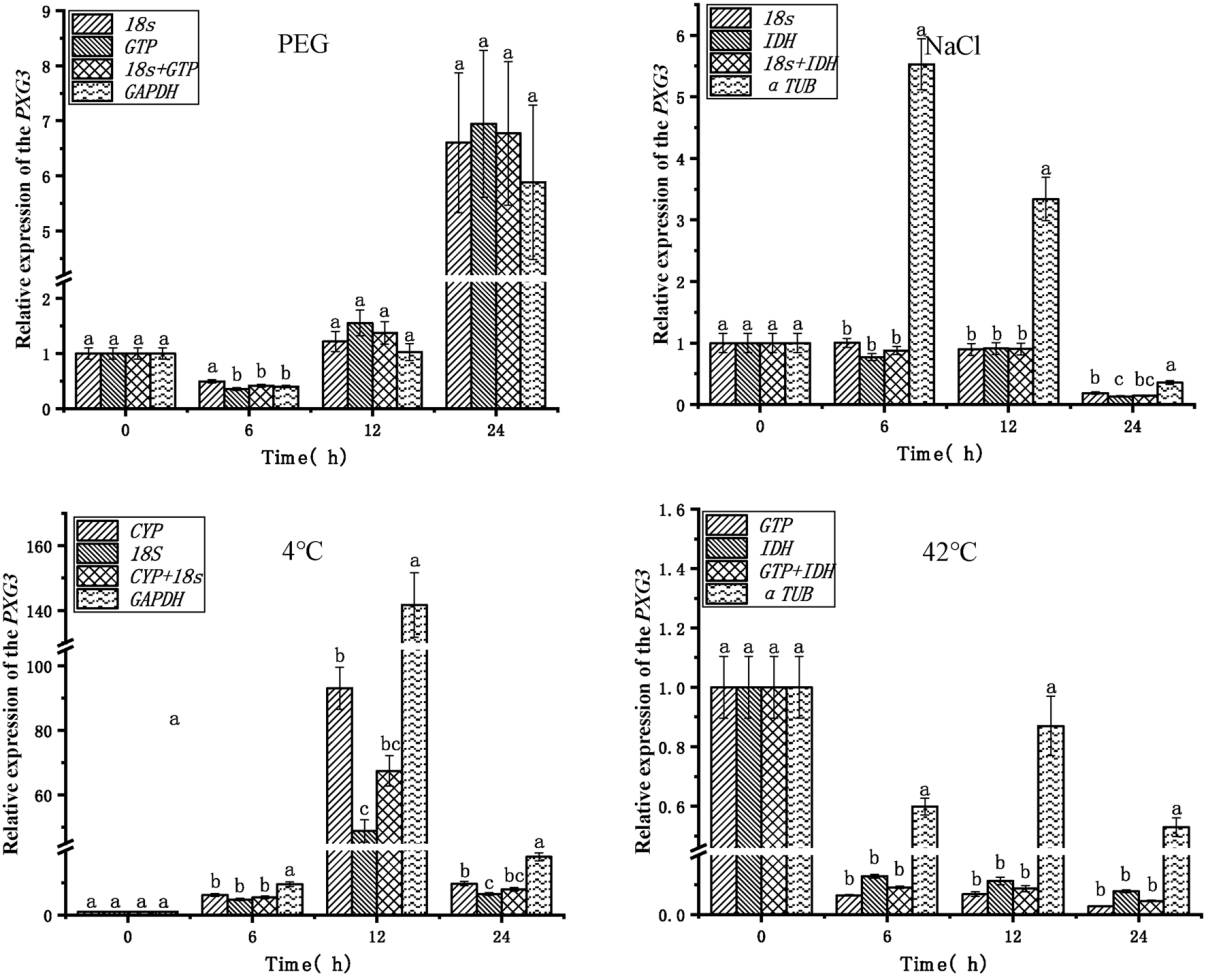
Relative expression of the *PXG3* target gene for validation of selected endogenous reference genes. *PEG* PEG-6000 drought treatment, *NaCl* 200 mM NaCl salt treatment, *4 °C* 4 °C cold treatment, *42 °C* 42 °C high temperature treatment. Different lowercase letters represent statistically significant differences as determined by one-way ANOVA (*P* < 0.05, Duncan’s multiple range tests).

To verify the reliability of the endogenous reference genes obtained in the above analysis, the expression patterns of the target genes *POD* and *PXG3* were analyzed in PEG, NaCl, 4 °C, and 42 °C stress treatments (Figs. 5, 6).The top two stable endogenous reference genes (*18S*, *GTP*), the combination of endogenous reference genes (*18S* + *GTP*), and the least stable endogenous reference gene (*GAPDH*) in PEG stress were used for normalization analysis, respectively. The expression trends of the target genes *POD* and *PXG3* were consistent, with the highest expression at 24 h. The expression of the *POD* gene was significantly different at 12 h and 24 h when *GAPDH* was used with *18S* + *GTP* and *18S*, *GTP*, respectively, as the endogenous reference genes. The most stable endogenous reference gene (*18S*, *IDH*), the combination of endogenous reference genes (*18S* + *IDH*), and the least stable endogenous reference gene (*α-TUB*) were used for normalization analysis in NaCl stress, respectively. The expression of the target genes *POD* and *PXG3* showed consistent trends and no significant differences in expression when the endogenous reference genes were *18S*, *IDH*, and *18S* + *IDH* (except *PXG3* at 24 h). However, the expression trends of *POD* and *PXG3* were inconsistent when *α-TUB* was used as an endogenous reference gene with *18S*, *IDH*, and *18S* + *IDH*, respectively, and the expressions were significantly different at 6 h, 12 h and 24 h. The endogenous reference genes with the highest stability ranking (*CYP*, *18S*), the combination of endogenous reference genes (*CYP* + *18S*), and the least stable endogenous reference gene (*GAPDH*) were used for normalization analysis in cold stress at 4 °C, respectively. The target gene *POD* expression trends were inconsistent except at 12 h. The expression trends of target genes *POD* and *PXG3* were consistent at other time points; meanwhile, the expression of *POD* and *PXG3* were significantly different when *GAPDH* was used as an endogenous reference gene with *18S*, *IDH*, and *18S* + *IDH*, respectively. The endogenous reference genes ranked first in stability (*GTP*, *IDH*), the combination of endogenous reference genes (*GTP* + *IDH*) and the least stable endogenous reference gene (*α-TUB*) were used for normalization analysis in heat stress at 42 °C, respectively; The target gene *POD* expression trends were inconsistent at 6 h, 12 h and 24 h. And the expression trend of *PXG3* was consistent. With *18S*, *IDH*, and *18S* + *IDH*, the expression trends of target genes *POD* and *PXG3* were consistent, and there was no significant difference in expression (except for *POD* at 12 h). In addition, the expression of *POD* and *PXG3* were significantly different when *α-TUB* was used as an endogenous reference gene with *GTP*, *IDH*, and *GTP* + *IDH*, respectively.

## Discussion

Using stable endogenous reference genes under different stress conditions is essential for the correct analysis, presentation, and interpretation of gene expression. We here analyzed the stability of genes using the commonly used endogenous reference gene screening software geNorm, NormFinder, Bestkeeper, and RefFinder software to determine the best endogenous reference genes for *Chlorella* sp. TLD 6B under different stress conditions. The results showed that the stability of these genes differed in different software evaluations.

The analysis with geNorm, NormFinder, and Bestkeeper under water, salt, low temperature, and high-temperature stresses, respectively, revealed that the ranking of *GTP* and *β-TUB* were very different, especially the scale of Bestkeeper with geNorm and NormFinder. This difference is mainly caused by these three methods^45–47^. We also found that most of the genes were ranked almost the same in geNorm and NormFinder, mainly due to the similar operating principles of GeNorm and NormFinder^45,46^. In this regard, the data from GeNorm, NormFinder and BestKeeper were evaluated comprehensively with RefFinder software to filter stable ones or genomic collaborations as endogenous reference gene^24,49^. In addition, GeNorm determines the number of required optimal genes based on V_n_/V_n+1_ values^50–52^. This feature of GeNorm has been utilized in most studies to identify the optimum number of endogenous reference gene. In this experiment, it was considered that two genes were required in drought and 42 °C treatment, while three endogenous reference genes were required in NaCl and 4 °C treatment.

These endogenous reference genes in this experiment showed different expression stability under various stress treatments. Among them, *18S*, *GTP*, *IDH* and *CYP* and their combinations were the most stable endogenous reference genes (Table 4). Several genes have been shown to be good stable samples, including *18S*^53,54^, *IDH*^55,56^, and *CYP*^57,58^. In addition, the experimental results showed that *GTP* can be used as a stable results which is contrary to Zhao’s results^41^; *GAPDH* and *α-TUB* were the least stable genes under various stresses in the present experimental catch, but were the most stable reference in some reported endogenous reference gene studies^59–61^, which is an indication that differences in species or experimental conditions lead to uniqueness of genes, therefore, the selection of suitable samples under different stress conditions is necessary to achieve reliable experimental results. The significant difference in GAPDH proteins under drought stress found in our previous study illustrates the instability of *GAPDH* genes in *Chlorella* sp. TLD 6B under abiotic stresses^62^.

The reliability of the stability of the endogenous reference genes was further verified by determining the expression patterns of the target genes *POD* and *PXG3* under drought, salt, and low and high-temperature stresses. The results showed that the expression patterns of target genes *POD* and *PXG3* under various stresses had obvious patterns of change when the most stable genes or combinations of endogenous reference genes were selected as controls according to different stress conditions. However, the expression patterns of the target genes *POD* and *PXG3* were significantly different or even showed different expression patterns between the most unstable endogenous reference gene, *GAPDH*, as the endogenous reference gene under drought and cold stress and *α-TUB* as the endogenous reference gene under salt and high temperature stress when the most stable endogenous reference gene or endogenous reference gene combination was selected (Fig. 4). This suggests that *GAPDH* is an unreliable endogenous reference gene in *Chlorella* sp. TLD 6B under drought and cold stress, and *α-TUB* is an unreliable endogenous reference gene under salt and high temperature stress. Similarly, it has been reported that the use of unstable endogenous reference genes as endogenous reference gene for RT-qPCR analysis detected significant differences in the expression levels of the target genes, leading to misinterpretation of the experimental results^52,63^. Therefore, selecting a suitable endogenous reference gene is important to normalize the target gene expression data generated by RT-qPCR.

## Conclusion

In summary, we evaluated the expression stability of nine endogenous reference genes in *Chlorella* sp. TLD 6B under four different abiotic stresses using RT-qPCR and four analytical software. We recommend using different endogenous reference genes when stress treatments are applied to *Chlorella* sp. TLD 6B. The *18S* and *GTP* are suitable for gene expression analysis under drought stress, while *18S* and *IDH* are the most stable genes in salt stress. *CYP* and *18S* maintain regular expression in low temperature stress, and the combination of *GTP* and *IDH* can be used as stable genes under high temperature stress. The results of this study will help to improve the accuracy of quantitative expression of target genes analyzed by RT-qPCR in Chlorella TLD6B under four common abiotic stresses, which not only promotes the functional genomics research of Chlorella desert, but also reveals the molecular mechanism of Chlorella TLD6B's rapid growth and adaptation to extreme desert environments, and is of great significance for its utilization and protection of soil desertification.

## Methods

### Experimental materials and treatment

The laboratory preserved *Chlorella* sp. TLD 6B was inoculated in BBM liquid medium for expansion. The expanded algae were re-inoculated into 200 mL triangular flasks in a 10:1 ratio and treated with abiotic stress after growth to the logarithmic growth phase (OD_680_ = 0.8). Among them, BBM medium with 15% PEG-6000 was used to simulate drought stress; BBM medium with 200 mM NaCl was used to simulate salt stress; and cold and heat stresses were carried out in artificial climate incubators at 4 °C and 42 °C, respectively. The Chlorella was collected at 0 h (ck), 6 h, 12 h and 24 h for each treatment, immediately snap-frozen in liquid nitrogen, and stored at − 80 °C. Three biological replicates were used for each stress treatment.

### Endogenous reference gene selection and primer design

According to the published *Chlorella* sp.TLD 6B transcriptome sequencing data (NCBI GEO: GSE162916), 9 endogenous reference genes (based on their FPKM and fold change values) were selected^24^. *Chlorella* sp. TLD 6B was evaluated by full-length transcriptome sequencing using PacBio instrument. Will get to the transcription of this sequence with TransDecoder after redundancy (https://github.com/TransDecoder/TransDecoder/Releases) to identify transcription candidate in this sequence coding region, *Chlorella* sp. TLD 6B CDS library is obtained. BLAST software (version 2.2.26) was used to compare the obtained non-redundant transcript sequences with NCBI non-redundant protein (NR), Swissprot (http://www.expasy.org/sprot/), GO (http://www.geneontology.org), COG(http://www.ncbi.nlm.nih.gov/COG/), Pfam (http://pfam.xfam.org/), KOG and KEGG databases (http://www.genome.jp/kegg) to obtain the annotation information of CDS database^64^. Primers for a total of nine genes, *18S*, *CYP*, *EF-1α*, *GAPDH*, *GTP*, *IDH*, *UBC*, *α-TUB*, and *β-TUB* were designed using Primer Premier 5(Premier, Canada) software according to Information about the CDS library and RT-qPCR primer design principles, and Sangon Biotech (Shanghai, China) synthesized the corresponding primers (Table 1).

### Total RNA isolation, cDNA synthesis, endogenous reference genes cloning, and RT-qPCR

The total RNA for each sample of *Chlorella* sp. TLD 6B was extracted with the RNAprep Pure Plant Kit (Tiangen Biotech, Beijing, China) according to the manufacturer’s protocol. RNA integrity was determined by 1.5% agarose gel electrophoresis, and RNA concentration and quality were determined by Nanodrop-2000. The cDNA was synthesized according to The cDNA was synthesized from 500 ng RNA according to the instructions of HiScriptIIl 1st Strand cDNA Synthesis Kit (Novozymes, Nanjing, China), with oligo (dT) primers in a final volume of 20 μL,and stored in the refrigerator at – 20 °C.

To test the specificity of each pair of primers, we first performed RT-PCR in a 50 μL system using a PCR instrument (Eppendorf AG, Hamburg, Germany), including cDNA 4 μL synthetic CDNA (100 ng), 2xPCR Mix 25 μL(Tiangen), 2 μL each of forward and reverse primer (10 μM), and ddH_2_O 17 μL. The amplification procedure consisted of an initial denaturation of 95 °C for 4 min, followed by denaturation at 95 °C for 30 s; annealing at 60 °C for 30 s; extension at 72 °C for 30 s in 30 cycles. The final extension at 72 °C for 5 min. The PCR products were detected by 2.0% agarose gel electrophoresis. They were recovered by agarose gel DNA recovery kit (Tiangen), and sequenced to verify the primers’ specificity. The RT-qPCR was performed using SYBR Green PCR Master Mix System (Novozymes) on an Applied Biosystems 7500/7500 Fast Real-Time PCR System (ABI, Foster City, CA, USA). In the reaction system of 20 μL: qPCR Master Mix (SYBR Green I) 10 μL, cDNA 1 μL (150 ng), forward primers and reverse primers 0.4 μL (10 μM) each, ddH_2_O 8.2 μL. Reaction conditions: initial denaturation.

95 °C for 3 min; denaturation at 95 °C for 10 s; annealing at 60 °C for 30 s; extension at 72 °C for 20 s in 40 cycles. The final extension at 72 °C for 5 min. Three biological and technical replicates for the sample of each treatment and tissue were used in the experiments.

### Stability assessment of endogenous reference gene expression

The stability of the endogenous reference gene was evaluated using GeNorm^46^, NormFinder^45^, BestKeeper^47^, and RefFinder^24^ software for the nine endogenous reference genes. For GeNorm and NormFinder software analysis^65^, the raw Ct values need to be converted into relative quantities according to the formula 2^−∆Ct^ (∆Ct = each corresponding Ct value—lowest Ct value). GeNorm was used to filter out the more stable endogenous reference genes by calculating the M value of stability of each gene. The criterion was that the smaller the M value, the better the stability of the gene. This program also evaluates the comparing pairwise variation (V_n_/V_n+1_) to determine the optimal number of genes required to normalize RT-qPCR data accurately. The V_n_/V_n+1_ threshold is generally considered 0.15, and if V_n_/V_n+1_ is less than 0.15, then the optimal number of endogenous reference genes is “n”^65^. The NormFinder program uses a model-based variance to calculate the stability value^45^, taking into account intra- and inter-group variations of the samples. A lowest value indicates the high expression stability of this gene^35^. For the BestKeeper program, the raw Ct values are used to calculate the coefficient of variation and standard deviation^27^. The principle of determination is that the greater the correlation coefficient, the smaller the standard deviation (SD) and the coefficient of variation (CV), and the better the stability of the endogenous reference gene. RefFinder program integrates the results of GeNorm, NormFinder, BestKeeper, generating a comprehensive endogenous reference gene stability ranking.

### Validtion of endogenous reference gene stability

It has been shown that *POD* and *PXG3* respond to abiotic stresses. To verify the reliability of the endogenous reference genes, *POD* and *PXG3* primers (Table1) were designed and further analyzed according to the RT-qPCR reaction system. The most stable and least stable endogenous reference genes were identified by RefFinder software, and the relative expression profiles of *POD* and *PXG3* in *Chlorella* sp. TLD 6B under abiotic stress were analyzed. Relative expression data were calculated for three biological replicates. Relative gene expression levels were calculated using the 2^−△△Ct^ method^66^.

### Statistical analysis

Statistical significance analysis of *POD* and *PXG3* was performed using IBM SPSS statistics 19.0 (https://www.ibm.com/products/spss-statistics) based on one-way ANOVA and further evaluated using Duncan’s multiple comparison (P < 0.05)^24^.

## Supplementary Information

Supplementary Figure S1.

## Data Availability

All data relevant to the study are included in the article or uploaded as supplementary file. In addition, the datasets used and/or analyzed during the current study are available from the corresponding author on reasonable request.
