# Supplementary material for: Screening and validating of endogenous reference genes in Chlorella sp. TLD 6B under abiotic stress
Source: Sci Rep. 2023 Jan 27;13:1555. doi: 10.1038/s41598-023-28311-x (PMC9883494; doi:10.1038/s41598-023-28311-x)
Supplement: Supplementary file 1 — Supplementary Figure S1. [file 41598_2023_28311_MOESM1_ESM.docx]

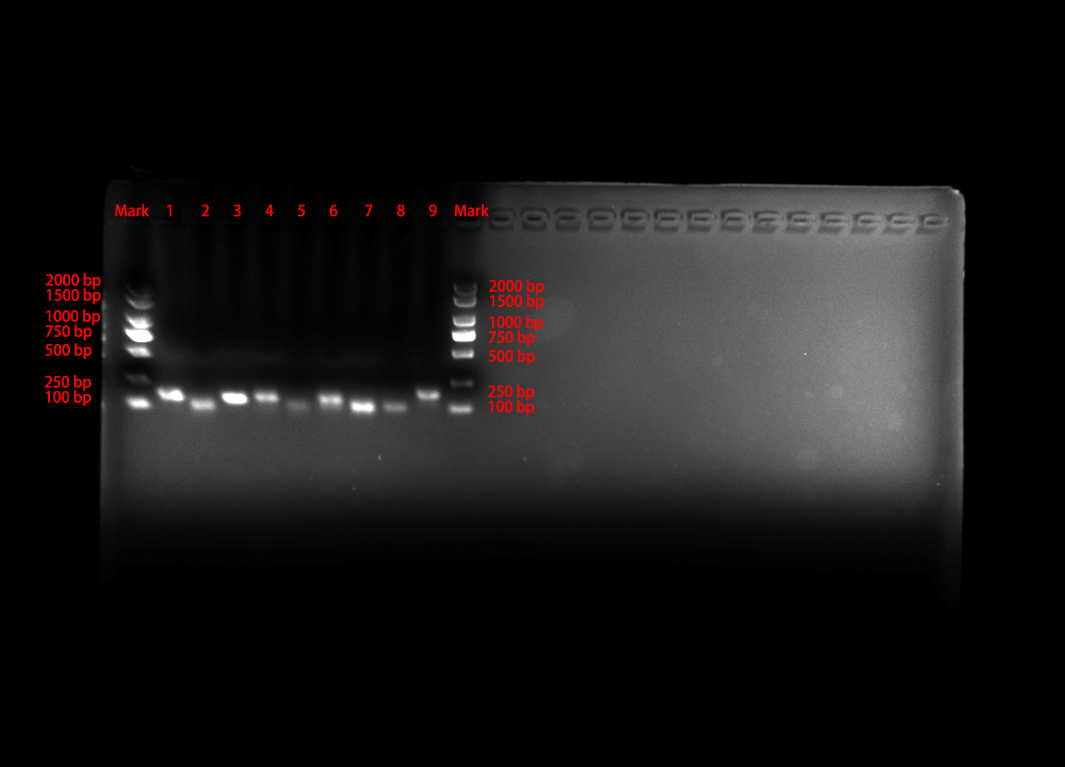


**Figure S1. RT-PCR amplification specificity.** Agarose gel (2.0%) electrophoresis indicated the amplification of a single PCR product of the expected size for 9 genes (lines 1–9: *18S*, *CYP*, *EF-1α*, *GAPDH*, *GTP*, *IDH*, *UBC*,*α-TUB*,*β-TUB*).
